# Tumour associated vasculature-on-a-chip for the evaluation of microbubble-mediated delivery of targeted liposomes†

**DOI:** 10.1039/d2lc00963c

**Published:** 2023-02-06

**Authors:** Matthew D. Bourn, Safoura Zahed Mohajerani, Georgia Mavria, Nicola Ingram, P. Louise Coletta, Stephen D. Evans, Sally A. Peyman

**Affiliations:** a School of Physics and Astronomy, University of Leeds Leeds LS2 9JT UK s.peyman@leeds.ac.uk +44 (0)1133433747; b Leeds Institute for Medical Research, Wellcome Trust Brenner Building, St James' University Hospital Leeds LS9 7TF UK

## Abstract

The vascular system is the primary route for the delivery of therapeutic drugs throughout the body and is an important barrier at the region of disease interest, such as a solid tumour. The development of complex 3D tumour cultures has progressed significantly in recent years however, the generation of perfusable vascularised tumour models still presents many challenges. This study presents a microfluidic-based vasculature system that can be induced to display properties of tumour-associated blood vessels without direct incorporation of tumour cells. Conditioning healthy endothelial–fibroblast cell vasculature co-cultures with media taken from tumour cell cultures was found to result in the formation of disorganised, tortuous networks which display characteristics consistent with those of tumour-associated vasculature. Integrin α*_v_*β_3_, a cell adhesion receptor associated with angiogenesis, was found to be upregulated in vasculature co-cultures conditioned with tumour cell media (TCM) – consistent with the reported α_*v*_β_3_ expression pattern in angiogenic tumour vasculature *in vivo*. Increased accumulation of liposomes (LSs) conjugated to antibodies against α_*v*_β_3_ was observed in TCM networks compared to non-conditioned networks, indicating α_*v*_β_3_ may be a potential target for the delivery of drugs specifically to tumour vasculature. Furthermore, the use of microbubbles (MBs) and ultrasound (US) to further enhance the delivery of LSs to TCM-conditioned vasculature was investigated. Quantification of fluorescent LS accumulation post-perfusion of the vascular network showed 3-fold increased accumulation with the use of MBs and US, suggesting that targeted LS delivery could be further improved with the use of locally administered MBs and US.

## Abstract 2

Increased accumulation of targeted liposomes was observed in cultures mimicking tumour-associated vasculature, when compared to healthy vasculature cultures. Microbubble-mediated liposome delivery was found to further increase liposome accumulation.

## Graphical abstract

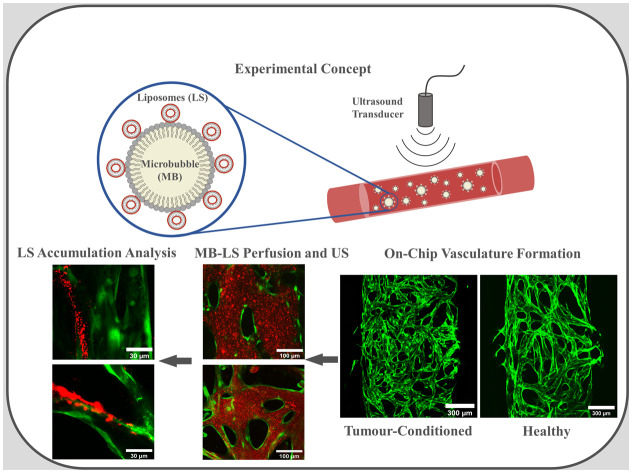

## Introduction

The successful development of increasingly complex microfluidic cell culture or organ-on-chip models has demonstrated the ability of these systems to effectively recreate the structure and function of many *in vivo* tissues. One area of particular interest is the development of microfluidic models that recapitulate the tumour vasculature and allow for the evaluation of anti-cancer therapeutics. Systems incorporating multiple healthy cell types alongside cancer cells have been developed and used to investigate a range of cancer-related processes, such as cancer cell response to chemical gradients and invasion into healthy tissues.^1–8^ Whilst endothelial cells have been incorporated into many tumour models, these are typically used in basic monolayer or pre-patterned configurations which do not accurately recreate the structure and function of tumour-associated vasculature.^9–11^ The endothelium presents the first barrier for therapeutics delivered intravenously and, as such, the absence of authentic vasculature components in tumour models often limits the validity of the test outcomes.

Recently, it has been discovered that vasculature networks can be produced by inducing the self-assembly of endothelial cells and fibroblasts into vessel structures in the presence of a continuous fluid flow.^12–14^ Endothelial cells and fibroblasts are seeded in a fibrin matrix into a microfluidic device, and then supplied with the appropriate growth factors to induce angiogenesis – the formation of new blood vessels. The co-culture of endothelial cells and fibroblasts recreates the process of vasculogenesis and angiogenesis that occurs at the site of wounds undergoing tissue repair and recapitulates the process of healthy network formation. Critically, the use of microfluidics overlaid in this process allows cells to be exposed to the physiological shear stresses and pressures required to induce vessel formation, whilst also allowing access to the vessel network by using connected side channels that mimic larger blood vessels. These vascularised organ-on-a-chip microfluidic systems present a promising platform for the investigation of many physiological processes and have recently been used for large-scale drug screening.^15^ However, without the influence of tumour cells, the vasculature produced in these systems will not display the necessary tumour-associated characteristics. The addition of tumour cells to vasculature cultures has allowed for the production of systems that recreate the *in vivo* responses of many anti-cancer therapeutics.^16^ However, the incorporation of tumour cells with healthy vasculature presents many challenges to ensure that the cultures progress in a similar fashion to tumours growing *in vivo.* Optimisation of seeding densities and timing is required to prevent tumour cells from overwhelming the healthy vasculature. Conversely, if cells are seeded too sparsely, then tumour development and influence over the vasculature will be limited.

The indirect influence of tumour cells has also been previously investigated as a means of inducing healthy cells to display tumour-associated characteristics. Katanasaka *et al.* used TCM taken from HT1080 cells to condition human umbilical vein endothelial cells (HUVECs) grown on Matrigel and found increased proliferation and angiogenic tube formation compared to vascular endothelial growth factor (VEGF) stimulated HUVECs. Analysis of protein expression found most differentially expressed proteins were downregulated (88%) with TCM, compared to an upregulation of 59% when VEGF was used to stimulate angiogenesis – revealing that the induction of tumour angiogenesis does not rely on VEGF alone.^17^ This highlighted the importance of using the appropriate tumour-derived angiogenic stimuli when seeking to recapitulate tumour angiogenesis. Hida *et al.* also investigated the effects of conditioning HUVECs with TCM and found that endothelial cells acquire drug-resistant characteristics when cultured in TCM. It was discovered that these characteristics are reversed when cells are re-cultured in TCM-free media.^18^ The presence of high levels of VEGF and other various inflammatory cytokines (CXCL1, 2) have been reported in media taken from patient-derived colorectal tumours and have been proposed as the main drivers of endothelial cell conditioning.^19^ Sawa-Wejksza *et al.* showed that media taken from several colorectal cell lines (HT29, LS180, SW948, SW620) could be used to induce THP-1 monocytes to differentiate into a non-adherent population of M1 and M2 immunosuppressive macrophages, consistent with tumour-associated macrophages found *in vivo*.^20^ These observations, along with various other studies,^21,22^ demonstrate that the cytokines and soluble factors tumour cells secrete, influence tumour-associated cells in a paracrine fashion, and can be used to condition cells to display similar properties to *in vivo* tumour-associated cells.

**Fig. 1:**
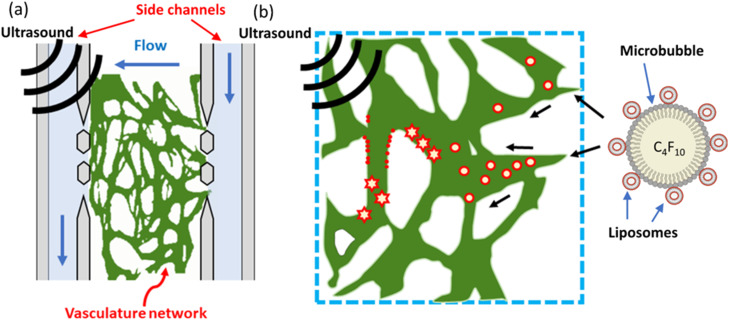
(a) A schematic illustrating the overall concept of the vasculature on-chip setup. A Centre chamber contains the vasculature network which is confined to the chamber with micropillars. The vasculature forms lumens at each micropillar gap which allows media from the adjacent side channels to perfuse through the network. Blue arrows depict the direction of flow throughout the system. (b) A close up depiction of microbubbles-mediated delivery of targeted liposomes to the vasculature. Liposomes produced using red fluorescent lipids (shown in red) are conjugated to the outside of microbubbles which are then perfused through the network. Ultrasound is then applied which bursts the microbubbles and facilities the deposition of liposomes onto the vasculature endothelium. Microbubbles, liposomes, and vasculature are not to scale.

This study combines the use of TCM conditioning and perfusable microfluidic vasculature models to develop a method of producing vasculature-on-chip networks that display tumour-associated properties. Fig. 1 shows a schematic of the microfluidic device and illustrates the overall concept of the vasculature-on-chip experiment design. A 3-channel design is used which consists of a central cell chamber containing the cell-fibrin suspension, flanked by media-containing side channels which provide both interstitial and intramural flow to vasculature cultures. The vasculature network forms in the centre chamber and lumens anastomose with the pillar gaps enabling perfusion of the networks. Liposomes are conjugated to the surface of microbubbles which are then perfused through the network. Ultrasound is then applied which bursts the microbubbles, facilitating the deposition and accumulation of liposomes on the vasculature endothelium.^2,23^ Further details regarding the choice of design features and experimental setup can be found throughout the materials and methods section.

By conditioning networks with media taken from tumour cell cultures, the soluble factors secreted by tumours induce vasculature to form similarly as if it were associated directly with tumour cells. Network functionality was demonstrated by using this system to evaluate the efficacy of targeting model drug carrier particles to integrin α_*v*_β_3_ – an integrin found to be upregulated in tumour vasculature. Integrin α*_v_*β_3_, an ECM-binding receptor found primarily on endothelial cells, has also been of recent interest due to its upregulation in tumour tissues. Integrin α_*v*_β_3_ binds to various matrix proteins found around newly forming blood vessels, such as fibrinogen, vitronectin and fibronectin.^24^ As a result, integrin α*_v_*β_3_ has been found to be crucial for angiogenesis and blocking of the signalling pathway has been found to reduce neovascularisation in tumour tissues.^25–27^ The constant pro-angiogenic signalling induced by tumour cells results in α_*v*_β_3_ being upregulated in tumour vasculature, presenting it as a potential target for drug-loaded carriers.^28,29^ Furthermore, integrin α_*v*_β_3_ upregulation in mammary fibroblasts conditioned with pro-inflammatory breast cancer factors has been previously reported, indicating that soluble factors secreted by tumour cells affect integrin expression.^30^

The use of microbubbles (MBs) to further facilitate the uptake of targeted therapeutics was tested to evaluate a combined localised delivery approach. MBs are micron-sized gas particles typically stabilised by a lipid shell. Due to the compressibility of their gas core, MBs are highly responsive to ultrasound (US) which causes bubbles to oscillate and scatter US waves.^31^ As a result, MBs have found use as US contrast agents in clinical imaging and have been used to facilitate the imaging of tumour vascular structures.^32–34^ MB also offer a potential means of enhancing drug delivery to cells through a mechanism known as sonoporation. Low acoustic pressure US waves can be used to induce MB to undergo stable cavitation, in which bubbles oscillate with small amplitudes at the US driving frequency. The rapid expansion and contraction of MBs exert forces on nearby cell membranes, creating pores (sonoporation) and increasing membrane permeability.^35–37^ Higher acoustic pressures can also be used to induce MBs to expand too much greater diameters then collapse and fragment into smaller particles. Significantly higher pressures and temperatures are generated when MBs collapse resulting in the generation of shockwaves. This process referred to as inertial cavitation has been associated with increased cellular uptake and increased irreversible sonoporation – where the pores created in cell membranes fail to reseal and result in cell death.^38,39^ Multiple *in vitro* studies have demonstrated the ability of sonoporation to increase drug delivery directly to tumour cells and endothelial cell-MB studies have been used to show increased permeability due to sonoporation, indicating that increased passage of therapeutics would occur.^40^ However, many of these investigations are carried out on 2D cell monolayers or in pre-patterned 3D configurations.^11,36,41,42^ Each of which neglect to recreate the authentic structure of vasculature which primarily consists of endothelial cells supported by a basement membrane layer and surrounding stromal cells.

The use of sonoporation as a means of facilitating the delivery of drug-loaded LS (LSs) is of particular interest for the treatment of cancer.^43,44^ Loading of anti-cancer therapeutics into LS has long been of interest and has been found to decrease systemic toxicity, allowing for higher drug doses to be tolerated.^45–47^ The conjugation of drug-loaded LSs to MBs to create therapeutic/drug-loaded MBs has been suggested as a potential means of delivering anti-cancer therapeutics locally to the tumour site, using US as a local release mechanism.^48^ If shown to be reliable and effective, this method of localised drug delivery to the tumour would overcome one of the major challenges anti-cancer treatments face – damage to healthy tissues from systemic drug exposures. The attachment of targeted antibodies to MB has also been explored as a method of increasing MB accumulation in tumour tissues – further increasing local drug-loaded LSs accumulation. Antibodies targeting VEGF pathway proteins have been of interest due to the upregulation of VEGF found in tumour-induced angiogenesis.^49,50^ A recent study by Ingram *et al.* observed the increased efficacy of cytotoxic drugs loaded into liposomes and attached to VEGFR2-targeted MBs. The US-triggered delivery was found to enhance localised tumour drug accumulation whilst simultaneously limiting the bioavailability of drugs in normal tissues.^51^ Integrin α_*v*_β_3_ targeting has also recently been tested and a study using α_*v*_β_3_-targeted MBs found that some sonoporation events induced endothelial cells to retract from neighbouring cells, resulting in the formation of a large gap in the endothelium.^36,39,50^ However, this investigation was conducted using a basic endothelial cell monolayer model – thus limiting the validity of these conclusions. The use of integrin α*_v_*β_3_-targeted MBs has been investigated as a means of imaging tumours and tumour angiogenesis using US.^52–56^ Anderson *et al.* observed a 5-fold increase in targeted MBs US intensity compared to controls and found significantly decreased intensity when mouse tumours were pre-treated with an antibody to block integrin α_*v*_β_3_.^57^ It is evident that α_*v*_β_3_-targeted MBs offer a promising means of targeting tumour vasculature, however further research into the impact of targeting on MB-mediated drug delivery is still required.

This study uses microfluidic vasculature cultures to investigate the efficacy of using α_*v*_β_3_-targeted LS and MBs to increase LS accumulation in tumour tissues. Networks mimicking tumour vasculature were created by growing vasculature in TCM taken from HCT116 colorectal cell cultures. Integrin α_*v*_β_3_ was observed to be upregulated in HUVECs and normal human lung fibroblasts (NHLFs) grown in TCM and was found to directly correlate with the concentration of TCM used. Networks conditioned with TCM were observed to undergo increased rates of angiogenesis, forming a disorganised network with larger diameter vessels. Targeted-LS were perfused through the networks and their accumulation in healthy and TCM-conditioned networks were first compared to evaluate the impact of α_*v*_β_3_ upregulation. Antibody targeting concentrations of 2 and 4 μg mL^−1^ were tested and compared to isotype controls of the same concentrations. Targeted LS were then conjugated to MBs which were perfused through the networks before being exposed to US. The efficacy of MB-mediated delivery of α_*v*_β_3_-targeted LS in TCM-conditioned networks was then evaluated and compared to those in healthy networks. This study reports a simple approach to condition on-chip vasculature to display disease-like characteristics and highlights the importance of producing accurate organ-on-chip models for the successful development of new therapeutics.

## Materials and methods

### Cell culture

Human umbilical vein endothelial cells (HUVEC) were purchased from TCS Cellworks (ZHC-2102) and grown using endothelial growth media-2 (EGM −2, Lonza). HUVECs were labelled at passage 1 with an enhanced green fluorescent protein (EGFP). Lentivirus production for the transduction was carried out following Tronolab protocols using HEK 293 T cells. 5 × 10^5^ HUVEC cells were plated in a T75 flask and incubated in virus-containing media for 16 hours. Fluorescent assisted cell sorting (FACS) was then used to sort transduced cells based on pre-determined gates based on the level of EGFP expression. Positive cells were reseeded and grown for a minimum of 48 hours to ensure sufficient viability. GFP-HUVECs were expanded to p3 before being frozen. Normal human lung fibroblasts (NHLF) were purchased from CellSystems and grown using FibroLife S2 (LifeLine Cell Technology) media. NHLFs were expanded to p3 and frozen. Both cell types were defrosted and grown for 2 days at p4, then used for the experimental setup.

### Microfluidic device and reservoir fabrication

Polydimethylsiloxane (PDMS) microfluidic devices were fabricated using standard photolithography and soft lithography techniques. SU8-2075 photoresist was spin-coated onto a 4 in. silicon wafer at 2000 rpm to produce a 100 μm thick layer. The photoresist was baked for 60 min then a direct-write 375 nm UV laser (DWL, Durham Magneto Optics) was used to pattern the device design by selectively exposing the photoresist. The wafer was then baked for a further 60 min then the excess photoresist was removed using Microposit EC solvent (Dow, US). PDMS (Sylgard 184, Dowsil) was mixed in a 10 : 1 base : curing agent ratio poured onto the wafer and desiccated for 40 min to remove any bubbles. PDMS was poured to produce 1 mm thick devices which minimised US attenuation through the PDMS. PDMS was then cured at 80 °C for 1 h. Devices were then cut, hole-punched and bonded to PDMS-coated coverslips using oxygen plasma.

Fabricated devices were then sterilised in an autoclave at 120 °C for 40 min. Reservoirs were fabricated from 16 mm diameter polycarbonate (Engineering and Design Plastics) cut into 32 mm long pieces. Reservoirs were fabricated to contain a 2 mm wide spout protruding from the bottom of each container. This allowed for simple interfacing with 2 mm hole-punched inlets and outlets in the microfluidic chips. Thicker pieces of PDMS were then bonded over inlet/outlet locations to secure reservoir interfacing with the chip. Reservoir lids were fabricated by gluing 0.22 μm PTFE filters (Cole-Parmer) onto the top of Delrin (Par-group) rings, which were then slotted over the top of the reservoirs. 0.22 μm filters prevented bacterial contamination of the media. Reservoirs were autoclaved at 120 °C for 40 min and the underside of the reservoir lids were sterilised under UV light for 30 min prior to experimental use.

### Microfluidic cell culture

Fibrinogen (Sigma, 341576-M) was prepared by dissolving 100 mg of power into 10 mL of sterile distilled water containing 0.9% NaCl and warmed to 37°. The solution was gently agitated until a cloudy solution was formed. The solution was then separated into 200 μL aliquots and frozen at −20 °C. Thrombin powder (100 U, Sigma, T4393) was dissolved in 2 mL of PDMS containing 0.1% w/v bovine serum albumin (BSA, Sigma, A9418) to give a 50 U mL^−1^ solution. The solution was then separated into 20 μL aliquots and frozen at −20 °C. Single aliquots of both fibrinogen and thrombin were defrosted prior to experimental use.

Both HUVEC and NHLF cells were detached from their culture flasks and resuspended together in fibrinogen at a concentration of 1 × 10^7^ cells per mL and 5 × 10^6^ cells per mL, respectively. 5 μL of the fibrinogen-cell mixture was then rapidly mixed with 0.5 μL of thrombin and pipetted into the centre chamber inlet of the microfluidic chip. Chips were then placed on a hotplate at 37 °C for 20 min to allow for fibrin polymerisation. Basement Membrane Matrix (BME, Cultrex, 3432-010-01) was then mixed with laminin (1 mg mL^−1^, Gibco, 23017015) and pipetted through the reservoirs, into the side channels. Chips were placed on the hotplate at 37 °C for a further 15 min to allow the BME mixture to partially polymerise. EGM-2 was then manually pipetted through each side channel to remove the majority of the BME mixture, leaving a coating remaining along the channel walls. Reservoirs were then filled with EGM-2 with 15 mmH_2_O and 5 mmH_2_O being added to inlet and outlet reservoirs, respectively. Flow direction was reversed daily to encourage complete anastomosis and media in each reservoir was releveled to ensure continuous flow and pressure differences. Fresh media was added every 48 hours to ensure consistent growth factor concentrations. EGM-2 complete with all growth factors was used for the first 24 hours before being replaced with growth factor reduced EGM-2 without VEGF or fibroblast growth factor (FGF), which was used throughout the rest of the experiment.

### AngioTool network morphology analysis

AngioTool^58^ is a software developed for the quantitative analysis of vasculature network morphologies from microscope images. Contrast and brightness enhancement was first applied to vasculature images to prevent misidentification of vessel structures. Multiple vessel diameters were selected for identification (10–150 μm in 5 μm increments) to ensure all vessels were correctly identified. The small particle removal maximum was set to 200 μm and the fill holes value set to 1200 μm, to ensure imaging artifacts and the intervascular space was correctly removed.

### Measurement of interstitial and hydrostatic flow velocity

The interstitial flow velocity through fibrin hydrogels was measured by perfusing 2 μM Calcein (Sigma-Aldrich, C0875) through blank fibrin gels produced using a range of fibrinogen concentrations (2.5, 5, 7.5 and 10 mg ml^−1^). On-chip flow conditions were setup as described above and the calcein solution added to the high-pressure side channel, whilst PBS was added to the low-pressure channel. Videos were captured of fluorescent calcein diffusion across the fibrin gels then FIJI (ImageJ) was used to analyse the velocity of the diffusion. Velocity values were expressed in μm s^−1^ and compared to optimal anastomosis interstitial flow values reported throughout literature. Fibrinogen concentrations which gave optimal interstitial flow values were then chosen for on-chip experiments.

Flow rates down each side channel, induced by the hydrostatic pressures from reservoir height differences, were measured by using 2 μm green, fluorescent beads (Thermo, FluoSpheres, F8853, 10^5^/ml). Beads were recorded flowing down the channels and their linear velocities were determined using Mosaic ImageJ particle tracking plugin (https://imagej.net/plugins/mosaicsuite). Linear velocities for reservoir height differences ranging between 2–20 mm were determined and compared to their theoretical values.

### Determination of vasculature permeability

The permeability of the vascular endothelium was determined by flowing Texas Red-Dextran (70 kDa, 100 μg ml^−1^, invitrogen) through the networks using the same on-chip flow conditions described previously. Perfusion was repeatedly imaged at multiple locations across the chip in 5 minute intervals and the leakage of the fluorescent dextran observed. The commonly implemented permeability model developed by Yuan *et al.* and further simplified by Lee *et al.* was then used to calculate vessel permeability using the known initial, final, and background fluorescent intensities.^59,60^

### HCT116 cell culture and TCM media preparation

HCT116 (ECACC 91091005) were obtained from ECACC (UK) and grown using DMEM 10% FBS (Thermo Fisher, UK), with 1% Glutamax (Gibco, UK), in an incubator at 37 °C, 5% CO_2_. Cells were STR profiled to ensure authenticity and regularly tested for mycoplasma. Approximately 10^6^ cells were seeded in a T-75 flask with 20 ml of media and allowed to grow to confluency for 5 days without a media change. TCM was then removed from the cell cultures and centrifuged at 15 000 × *g* for 15 minutes to remove any cell debris. TCM was mixed with endothelial basal media, EBM (EGM-2 without the supplementary Lonza SingleQuots growth factor kit added) to the desired TCM : EGM ratio ([2 : 1], [1 : 1], and [1 : 2]). EGM-2 supplementary growth factors were then added to ensure the overall concentration remained constant.

### Off-chip cell culture seeding

HUVEC and NHLF cells were seeded off-chip in a similar configuration as on-chip vasculature cultures. 1 × 10^4^ NHLF cells were first seeded in Fibrolife S2 media in each well of a 24-well plate then allowed to grow to form a confluent monolayer for 96 hours. HUVEC cells (1 × 10^7^ cells per mL) were then suspended in fibrinogen (7.5 mg mL^−1^) and mixed with thrombin (50 U mL^−1^) to create the same fibrin-cell suspension used on-chip. 10 μL of this suspension was then seeded directly onto the NHLF monolayer. These cultures were grown in EGM : TCM media ratios of [1 : 0], [2 : 1], [1 : 1], and [1 : 2] for a further 96 hours at which point immunostaining and flow cytometry was used to determine α_*v*_β_3_ -expression in each of the culture conditions.

### Immunostaining and flow cytometry

Cell-containing fibrin hydrogel clots were first digested using nattokinase (50 FU mL^−1^) for 60 minutes. Cells were then detached, washed, and resuspended in a solution of PBS (10% FBS) and anti- α_*v*_β_3_ antibody (Abcam, ab190147) diluted 1 : 2000 with PBS (3% BSA). Cells were incubated for 30 minutes then centrifuged at 300 g for 5 minutes and washed 3 times with PBS. Cells were then resuspended in Alexa Fluor 647 secondary antibody (Abcam, ab150115) diluted 1:2000 with PBS (3% BSA) and incubated in the dark for a further 30 minutes. Cells were again centrifuged at 300 g for 5 minutes and washed in PBS 3 × then resuspended to a final concentration of 1 × 10^6^ cells per mL in PBS (10% FBS). Flow cytometry was performed by Dr Ruth Hughes and Dr Sally Boxall in the Bioimaging and FACS facility within the faculty of Biological Sciences at the University of Leeds. A CytoFLEX S (Beckman Coulter) cytometer was used to excite GFP-HUVECs and the Alexa Fluor 647 secondary antibody with 488 nm and 638 nm lasers, respectively. A minimum of 10 000 valid events were captured for each sample to allow for robust data analysis. Fluorescence minus one compensation controls were first performed using α_*v*_β_3_-stained NHLFs and GFP-HUVECs as red-only and green-only controls, respectively. Unstained NHLFs were also used as double-negative controls. Flow cytometry data was gated with respect to these controls to determine the number of α_*v*_β_3_-positive cells. Immunofluorescent intensities were plotted as histograms and a Lorentzian peak fitting function used to find the modal peak intensity for each sample. Immunostaining of microfluidic vasculature cultures was performed by perfusing the networks with PBS (+Ca and +Mg) for 30 minutes Comment using the same on-chip growth flow conditions. Cultures were then fixed with 4% paraformaldehyde for 90 minutes which was followed by a 60 minute PBS wash. Cultures were permeabilised by flowing 0.3% Triton-X100 for 30 minutes then a blocking solution of PBS (3% BSA, 0.3 M Glycine, 0.1% Triton-X100) was perfused for 60 minutes. The primary antibody (Abcam, ab190147) was diluted 1 : 1000 in PBS (3% BSA) and perfused through the network overnight at 4 °C. A 60 minute PBS wash was then performed then the secondary antibody (Abcam, ab150115) diluted 1 : 1000 in PBS (3% BSA) was perfused for 3 hours. A final 90 minute PBS wash was performed then the cultures were imaged using a Leica DMi8/SP8 confocal microscope.

### Targeted liposome and microbubble production

LS were prepared from a mixture of DSPC (Avanti Lipids, 850365), Cholesterol (Avanti Lipids, 700100), DSPE-PEG2000 Biotin (Avanti Lipids, 880129) and DOPE-647 N (Sigma-Aldrich 42247) in a 55 : 40 : 4.6 : 0.4 M ratio with a total lipid concentration of 12 mg mL^−1^. Lipids were dissolved in a 1 : 1 mixture of chloroform and methanol and dried under nitrogen to remove the solvent. Lipids were then resuspended in PBS and heated at 65 °C. LS were produced *via* extrusion through a 400 nm polycarbonate filter membrane (Avanti Lipids, 610007). LS concentration was determined using a NanoSight NS300 (Malvern Panalytical) and was typically found to be between 2–4 × 10^12^ LS mL^−1^. The average LS diameter was determined to be 335 ± 65 nm, found using a Zetasizer (Malvern Panalytical). Biotin – avidin binding was used to conjugate α_*v*_β_3_ – antibodies to the surface of the LS. Biotin α_*v*_β_3_ antibody (Biolegend 30412, 0.5 mg mL^−1^) was mixed with LS (10^11^ mL^−1^) to give a final concentration of 2 or 4 μg mL^−1^. Neutravidin (Thermo Scientific, 31000) was then added, the solution mixed, and the antibodies allowed to bind for 20 minutes before perfusion. The same protocol was used for biotinylated isotype antibody controls (Biolegend 400 103, 0.5 μg mL^−1^). 3 kDa cascade blue dextran (invitrogen, 0.1 mg mL^−1^) was also added to the LS solution to allow for perfusion through the networks to be imaged alongside LS perfusion.

MBs were prepared from a mixture of DPPC and DPSE-Biotin-PEG2000 in a 95 : 5 M ratio and a total lipid concentration of 2 mg mL^−1^. Lipids were dissolved in a 1 : 1 mixture of chloroform and methanol and dried under nitrogen to remove the solvent. Lipids were then resuspended in PBS solution containing 1% glycerol and tip sonicated (20 kHz, 150 W, Sonifier 250, Branson, USA) at 4 °C for 40 min. The lipid solution was transferred to a 1.75 mL glass vial (Ampulla, UK) C_4_F_10_ gas (F2 chemicals) was then bubbled through the lipid solution for 2 minutes using a pressure of 200 mbar. Immediately after bubbling, the polypropylene cap was replaced, secured with parafilm, then placed in a shaker (VialMix, Bristol Myers Squibb) for 45 seconds. After MB production, size and concentration distribution were measured optically using bright-field microscopy and analysed using a custom MATLAB (2017b, MathWorks, USA) script. MBs were typically produced at a concentration between 3–7 × 10^9^ MB mL^−1^ with an average diameter between 1 and 2 μm. MBs were mixed with targeted-LS for a final MB : LS ratio of 10^9^ : 10^11^ and allowed to bind for 20 minutes on ice prior to perfusion. This previous ratio of MB : LS : Neutravidin has been previously shown to produce MB–LS conjugates using fluorescent microscopy to observe the association of fluorescent LSs to the MB surface^40^ and has also been used to produce targeted-LS–MB complexes in related literature.^51^

### Liposome/microbubble perfusion and imaging

The inlet reservoir for perfusion was first filled with 2.2 mL of media and the corresponding outlet filled with 0.5 mL. The inlet and outlet reservoirs in the adjacent media channel were filled with 0.3 mL and 0.1 mL, respectively. This configuration maximised the interstitial pressure across the vasculature chamber, maximising the intramural flow through the network. Once the flow was initiated, 30 μL of LSs, MBs, or LS–MBs were deposited into the bottom of the inlet reservoir and allowed to perfuse through the network for 1 hour. Perfusion through the network was imaged using a Leica DMi8/SP8 confocal microscope, with the GFP and atto647N being excited with 488 nm and 638 nm diode lasers, respectively. The mark and find feature was used to periodically image the network in 5 minutes intervals at multiple (typically 8–10) locations to observe LS, MB, and dextran perfusion across the network. Microfluidic devices were insonated once MBs or LS–MBs were observed to have populated the entire network. 1 hour after perfusion began, fresh media was added to the inlet reservoir and allowed to perfuse through the network for an additional hour. This rinsed all remaining LS solution from the network, ensuring that all of the remaining LS were those which had accumulated within the vasculature. Post-perfusion images were then taken using a 100× objective, which captured confocal *z*-stacks using a 1.5 μm slice thickness.

### Ultrasound instrumentation and exposure

Microfluidic devices were insonated using a 2.25 MHz centre frequency unfocussed transducer with a 6 mm element diameter (V323-SM, Olympus, US). Ultrasound pulses were controlled by a function generator (TG5011, Agilent Technologies, UK) and consisted of a duty cycle of 1%, a pulse repetition frequency of 1 kHz for a total duration of 2 seconds. The free field transducer output was previously calibrated as described here and found to produce a peak negative pressure of 0.81 ± 0.04 MPa when driven by a +546 dB RF power amplifier (A150, Electronics & Innovation, US). This produced a mechanical index (MI) of 0.54 ± 0.03. The transducer was coupled to the top of the microfluidic device at a 180° angle *via* a 2 cm thick gel standoff pad (Aquaflex, Parker Laboratories, US) and coupling gel to ensure only far-field, uniform US waves reached the device. These US parameters have been previously used in on-chip MB-related experiments and have been shown to burst approximately 99.6% of MBs contained within a PDMS device with a 1 mm wall thickness.^40^ Characterisation of US attenuation through 1 mm of PDMS has been shown to reduce peak negative pressure to 0.81 ± 0.04 MPa, a 16% reduction from the 0.94 ± 0.01 MPa pressure measured through degassed ultrapure water. This resulted in a mechanical index (M.I) value of 0.54 ± 0.03.

### Quantification of liposome accumulation

Post-perfusion *z*-stack images of LS fluorescence were used to quantify the amount of accumulation within the vasculature networks. ImageJ particle analysis tool was used to identify LS and quantify their fluorescent intensity. Contrast enhancement was first performed on captured images to reduce any background fluorescence and ensure that LSs were correctly identified. *Z*-Stacks were duplicated, and the duplicates were binarised using the threshold tool. Thresholding values were determined using images of vasculature which had not been perfused with LSs. Measurements were set to the original stack, using the binarized stack as a mask for the original stack. The analyse particles tool was then used to quantify LS fluorescence. The values from each stack of images were averaged and plotted as a single data point on a boxplot graph. Imaging locations were kept relatively consistent across each microfluidic chip with the choice of locations spanning the area of vasculature network. Between 5–8 *z*-stacks were taken for each microfluidic chip used, depending on time constraints. Each set of accumulation data was capture from 3–7 separate microfluidic chips used across a minimum of 3 separate experiments. Accumulation data was then subject to a Mann–Whitney non-parametric statistical test which ranks data by value then determines the *P* value by the number of times a point from one dataset is higher than a point from another.

## Results and discussion

### Microfluidic device design and characterisation

**Fig. 2:**
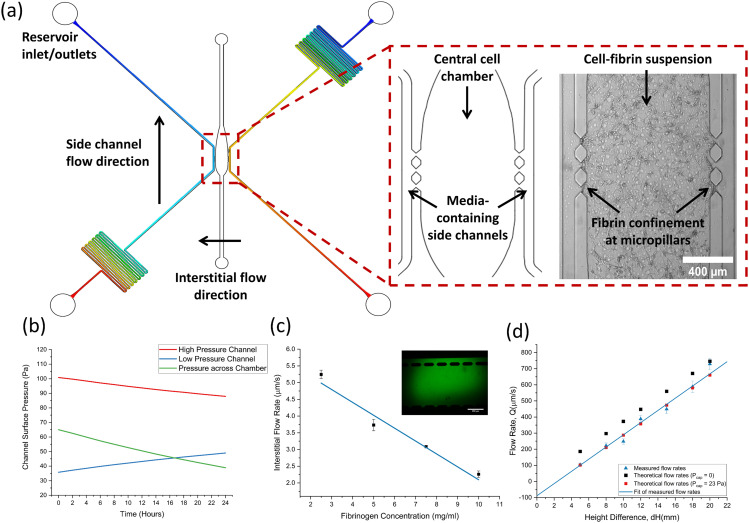
(a) An AutoCAD outline of the microfluidic device with pressure distribution plots overlayed on the side channels to display pressure equilibration between reservoirs along the channel. The inset shows a close-up of the three-chamber design in AutoCAD, alongside a brightfield image taken after the cell-fibrin suspension was seeded into the central chamber. (b) Graph showing the surface pressure at the pillar gaps in the high- and low-pressure side channels as a function of time. The pressure across the chamber slowly decreases due to decreasing hydrostatic flow. (c) A graph of interstitial flow through empty fibrin gels formed with a range of fibrinogen concentrations. (d) Measured and theoretical flow rates for a range of reservoir height differences. Red data points show theoretical flow rates once the measured capillary pressure value was included in calculations.

Fig. 1a shows a computer aided design (CAD) schematic of the microfluidic device alongside a close-up of the central cell chamber. The microfluidic device used throughout this study features a 3-channel design consisting of a wider central cell culture chamber, flanked by two narrower side channels for media flow. Micropillars are used to separate the three channels, enabling the confinement of cell-laden hydrogels in the centre chamber whilst still allowing for diffusion of nutrients from the media channels. As the pressure gradient differs along the length of the channel, a limited number of micropillars were included in the centre of each chamber to prevent significant pressure differentials. Along with the design of the centre chamber and micropillars, careful consideration of media channel dimensions was required to successfully emulate physiological rates of flow. Flow down the channel was induced by hydrostatic pressure through the use of media reservoirs interfaced into the side channel inlets and outlet. The magnitude of the hydraulic resistance, *R*_hyd_ is dictated by channel length, *L*, and the overall flow rate is dictated by the height difference, Δ*H*. Serpentine side channels were therefore used to increase the hydraulic resistance down the channel and decrease flow rates to physiological levels. Further information on the equations governing hydrostatic flow can be found in the supplementary material. The pressure distribution along each channel, shown in Fig. 2a, was also an important parameter to consider, as interstitial flow rates across the chamber dictate the morphology of the vasculature network formed. Furthermore, interstitial flow across the chamber becomes intramural flow once a perfusable network has formed – meaning pressure differences across the chamber must also be sufficient to enable physiological rates of flow throughout the vasculature network.

Side channels were designed to produce flow rates ranging from 100–800 μm s^−1^ depending on the relative difference in reservoir fluid levels which could range up to Δ*H* = 22 mmH_2_O. The asymmetric positioning of serpentines allowed for a pressure differential to be created across the centre chamber whilst maintaining equal rates of flow down each channel. Fig. 2b shows theoretical modelling of the channel pressures at the micropillar region change as a function of time, modelled using initial inlet and outlet reservoir heights of 12 mm and 2 mm, respectively. The pressure across the channel decreases with time due to the emptying of inlet reservoirs. Interestingly, pressure in the low-pressure channel increases with time due to the filling of the outlet reservoir, which increases the backpressure at the pillars due to the short channel distance.

The effect of this pressure differential was observed by measuring the interstitial flow across the channel by imaging the diffusion of Calcein across a blank fibrin gel. Fig. 2c shows the rate of interstitial flow through fibrin gels made with differing fibrinogen concentrations, alongside an insert showing typical green, fluorescent Calcein diffusion across the gel. Flow rates were observed to decrease as fibrin concentration increased and followed a linear trend as shown by the fitted line. Flow rates ranged between 2–5.2 μm s^−1^, which is well within the range of flow rates reported to be optimal in inducing angiogenesis and anastomosis (0.1–6 μm s^−1^).^61^ From these observations, it was decided that a final fibrinogen working concentration of 7.5 mg mL^−1^ would be used to form the fibrin gels. This provided a sufficiently dense scaffold for cells to interact with whilst allowing for sufficient interstitial flow and nutrient exchange.

Flow through the side channels was characterised by first filling the centre chamber with a blank fibrin gel (7.5 mg mL^−1^ fibrinogen) then perfusing and tracking the trajectory of 2 μm green, fluorescent beads down a side channel. Fig. 2d shows the measured flow rates down a side channel plotted alongside theoretical flow rates for a range of reservoir height differences. As expected, the trend for both theoretical and measured flow rates shows an increase in flow rate with increasing height difference. The measured hydraulic resistance was found to be (2.4 ± 0.1) × 10^13^, agreeing closely with the theoretical value of 2.6 × 10^13^. Theoretical flow rates were initially plotted using a capillary pressure, *P*_cap_ = 0, which fixes the intercept to the origin. However, the intercept from measured flow rates allowed *P*_cap_ to be calculated and found to be 23 ± 5 Pa. Theoretical flow rates were replotted using this value to demonstrate the agreement with measured flow rates. Overall, it was shown that physiological rates of flow could be provided using hydrostatic pressure and reservoirs could provide reasonable flow rates for up to 24 hours, at which point fluids were re-levelled and the initial flow rate restored.

### Tumour vasculature production and characterisation

**Fig. 3:**
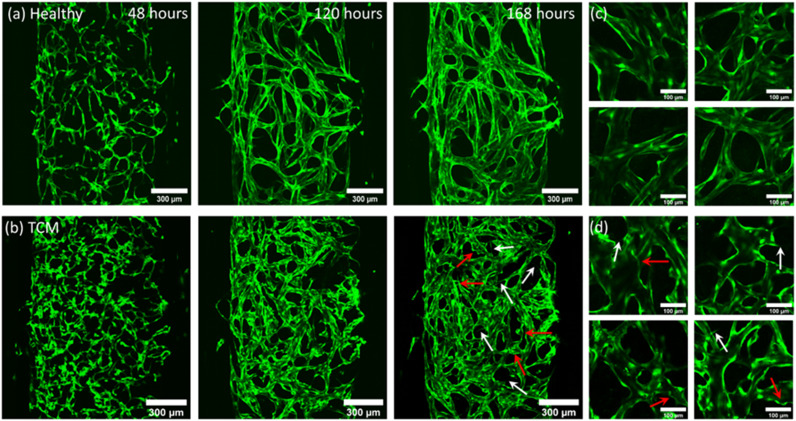
Fluorescent images acquired (using 10× objective) of GFH-HUVECs self-assembling into fully perfusable vasculature networks 48, 120 and 168 hours after seeding. (a) Shows healthy cultures grown using EGM-2 media and (b) shows cultures conditioned with [2 : 1] EGM : TCM media, initiated after 48 hours of culture in EGM-2. White arrows highlight branching structures and red arrows highlight tortuous, bulbous vessels morphologies. (c) and (d) Show higher magnification images of healthy and TCM networks taken from several networks used in this study, respectively. Images show TCM conditioned networks produce a fragmented, tortuous vasculature network compared to the smooth, continuous networks formed with regular EGM-2 media.

GFP-HUVECs and NHLFs were seeded in a fibrin matrix and induced to self-assemble into a vasculature network using EGM-2 media. Healthy networks were formed using complete EGM-2 for the first 24 hours, then growth factor reduced EGM-2 without VEGF and FGF used thereafter. Removing VEGF and FGF from the media, induced vessel maturation and lumen formation and allowed HUVECs to become dependent on the growth factors secreted by the NHLFs, rather than exogenous stimuli. Networks were induced to display tumour vasculature characteristics through the use of TCM. Networks were grown using EGM-2 to allow cultures to become established, then, after 48 hours, the growth factor reduced EGM-2 media was replaced with media containing growth factor reduced EGM-2 and TCM in a [2 : 1] ratio. Fig. 3 shows confocal images taken of GFP-HUVECs self-assembling into (a) healthy (EGM-2 only) and (b) TCM ([2 : 1] EGM : TCM media) networks. Images were taken at 48, 120 and 168 hour time points, after which time the networks had undergone sufficient lumen formation and were able to be perfused and suitable for experimental use. High magnification images of (c) healthy (EGM-2 only) and (d) TCM ([2 : 1] EGM : TCM media) networks can also be observed in Fig. 3, which enables the morphological differences between networks to be further observed. Growing vasculature networks in higher EGM : TCM ratios, such as [1 : 1] and [1 : 2] was explored however, the use of higher concentrations resulted in the formation of networks with extreme morphologies which could not be reliably perfused (ESI† Fig. S1). In many instances, high TCM concentrations in [1 : 2] EGM : TCM ratios, were observed to induce the contraction of vasculature cultures away from the microfluidic chamber walls. This was theorised to either be a result of fibroblasts becoming cancer-associated and displaying increased contractile characteristics, or a consequence of large vessel diameters resulting in the collapse of vessels.

Cells were observed to elongate to form a continuous branched network across the first 96 hours, at which point they begin expanding to form lumenised vessels. Up to 48 hours, there was no difference between the networks in the EGM-2 group and [2 : 1] EGM : TCM-group as growth conditions were the same. After [2 : 1] EGM : TCM media was added at 48 hours, morphological differences became apparent at the 120 hour time-point. [2 : 1] EGM : TCM networks were found to initially form more fragmented networks with continually branching structures protruding from vessels which are highlighted by white arrows in Fig. 3b (168 hours). In comparison, healthy networks formed continuous networks more readily and showed little branching after 120 hours. Healthy networks were observed to mature after 144 hours, at which point lumen formation stabilised and perfusion was possible. Whereas vasculature formed using [2 : 1] EGM : TCM media was not observed to stabilise, vessels continued to form additional branches and wider lumens. This was reflected in images taken after 168 hours, which show healthy networks having a smooth consistent morphology compared to [2 : 1] EGM : TCM networks which have disjointed, tortuous structures which are highlighted by red arrows in Fig. 2b (168 hours). Networks formed with TCM conditioning showed some characteristics similar to those formed with EGM-2 media without VEGF and FGF growth factors removed (ESI† Fig. S2). The constant pro-angiogenic VEGF and FGF stimuli ultimately resulted in networks failing to form realistic structures, but the rounded vessel morphologies were like structures observed in TCM networks. This indicated that the use of [2 : 1] EGM : TCM induced networks to undergo continued angiogenesis, inhibiting vessel maturation and driving the overgrowth of vessel lumens. This agrees with observations from previous studies which have found high levels of VEGF in media taken from tumour cells.^19^

**Fig. 4:**
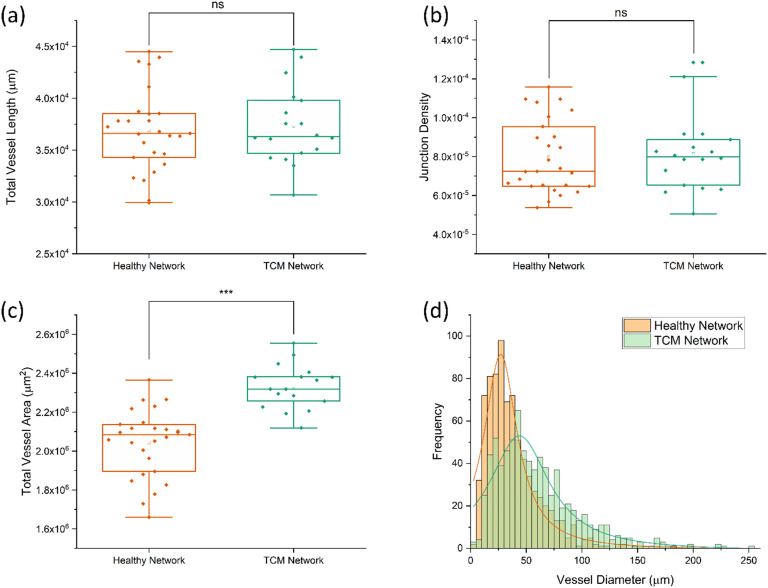
Boxplots of total vessel length (a), junction density (b), and total vessel area (c) for healthy and TCM networks, determined using AngioTool image analysis. Each data point corresponds to a single network. Comparison of healthy and TCM networks found no statistical difference between total vessel length or junction density. Total vessel are was consistently larger for TCM networks, resulting a 14% increase on average. (d) Histogram plot of vessel diameters measured using ImageJ. Lorentzian fits used to find modal diameter peak, corresponding to 24.5 ± 1.1 μm (*R*-square = 0.92) for healthy networks and 37.5 ± 2.3 μm (*R*-square = 0.83) for TCM conditioned networks.

The morphological effects of [2 : 1] EGM : TCM conditioning were quantified by analysing networks using AngioTool^58^ – giving values for junction density, total vessel length and total vessel area. Vessel diameters were also measured using ImageJ and plotted as a histogram. Fig. 4 shows the resultant plots comparing healthy (EGM only) and [2 : 1] EGM : TCM network morphologies. AngioTool analysis revealed no significant difference in values for total vessel length (Fig. 4a) and junction density (Fig. 4b) whereas total vessel area (Fig. 4c) was observed to increase significantly in [2 : 1] EGM : TCM networks. Healthy networks were found to have a total vessel area of (2.0 ± 0.2) × 10^6^ μm^2^ and TCM networks (2.3 ± 0.1) × 10^6^ μm^2^ – a 14% increase. The effects of TCM conditioning were further shown when vessel diameters were analysed which is shown in the histogram in Fig. 4d. The increased lumen formation induced by the apparent pro-angiogenic signalling from TCM media resulted in the peak median vessel diameter increasing approximately 50% – from 24.5 ± 1.1 μm in healthy networks to 37.5 ± 2.3 μm in TCM networks. This was primarily influenced by the observation of significantly more vessels exceeding a diameter of 50 μm. Observations of increased vessel dilation, overall area and fragmented structures are well aligned with observations throughout previous tumour vasculature models. Eddy and Casarett studied the development of tumour vasculature in a malignant neurilemoma hamster model using a transparent cheek pouch chamber.^62^ Vessels close to the tumour implant were found to have dilated, increased in tortuosity, and formed bulbous structures. Angiogenic sprouting from host vessels was also observed and resulted in remodelling of the host venules as the tumour increased in size. Tumour vessels were observed to reach diameters as large as 200 μm, consistent with maximum diameters observed throughout this system. The effect of tumours on vasculature morphology was also investigated by Yamaura and Sato, who observed a 3-fold increase in vascular surface area and a 5-fold increase in vascular length, per unit volume of tissue.^63,64^ Together, these observations support those made throughout this study, and further support the supposition that TCM can indirectly induce a similar tumour vasculature morphology to vasculature produced through direct contact with tumour cells.

Network functionality and endothelial barrier function were demonstrated by perfusing Texas-Red Dextran (70 kDa) through the network. This allowed for observation of flow through the network and subsequent calculation of the permeability coefficient using the rate of leakage through the vessel walls. Perfusion across a series of locations was imaged periodically for 45 minutes (ESI† Fig. S3) and the permeability coefficient, *P*_D_ calculated using a commonly used permeability model for vasculature networks.^59,60^ From this, *P*_D_ was calculated to be (3.9 ± 0.6) × 10^−7^ cm s^−1^ showing good agreement with previous values throughout literature, which typically quote permeability values between (1.2–15.5) × 10^−7^ cm s^−1^.^16,60,65^ Observation of an effective endothelial barrier by measurement of dextran perfusion exclusively through the vasculature gave confidence in this system's ability to effectively recreate functional vasculature networks. Flow through networks was further characterised by tracking the perfusion of 2 μm green, fluorescent beads through the vasculature (shown in ESI† Fig. S4). Intramural flow throughout the network was maximised by adding 2.2 mL of solution to the high-pressure inlet reservoir and 0.5 mL to the outlet reservoir in the same channel. Adjacent reservoirs were filled with 0.1 mL of solution to maximise the pressure difference across the chamber. The average flow rate was found to be 91 ± 10 μm s^−1^, with frame-to-frame velocities found to range between 16–230 μm s^−1^. Bead trajectories and speeds were found to vary significantly as they traversed the networks, this was due to the differing vessel diameters affecting hydraulic resistance and in turn, flow rate. Flow rates through capillary networks are typically found to be between 100–1000 μm s^−1^, meaning the average value in this system is slightly lower than generally observed.^61,66^ The primary effect of this is the reduction in shear stress experienced on the vessel surface. Using the basic model for shear stress induced by laminar flow through a cylindrical pipe, the shear stress is estimated to be 0.92 ± 0.1 dyne per cm^2^, whereas physiological shear stresses within capillaries are found to range between 1–100 dyne per cm^2^. The lower flow rates along with the differing viscosities between media and blood result in lower than desired shear stress values across this network. There is no doubt that the range shear stress experienced across the network will fall within physiological values, as it is the only average value that is slightly lower. Overall, the shear stress value may influence the accumulation of targeted LS within the networks, as adhesion to the vessel surface will be affected. However, this was not deemed to be a major concern as shear stress was only 10% lower than desired, and accumulation results would all be measured relative to non-targeted and isotype controls.

### Tumour cell media-induced upregulation of integrin α_*v*_β_3_

The pro-angiogenic effects of [2 : 1] EGM : TCM conditioning on network morphology has been observed in the previous on-chip vasculature experiments. To quantitatively assess the effect of TCM conditioning on HUVEC-NHLF fibrin cultures, immunostaining and flow cytometry was used to determine how integrin α*_v_*β_3_ expression is influenced by the presence of TCM. NHLFs and HUVECs were grown in an off-chip, 24-well plate configuration which closely recreates the on-chip seeding configuration of vasculature cultures. This seeding configuration is also based on a common 2D branching assay used to evaluate the angiogenic ability of endothelial cells. Culturing cells off-chip allowed for straightforward immunostaining, detachment, and flow cytometry analysis, compared to cells grown on-chip which were not readily retrievable from the microfluidic chamber.

ESI† Fig. S5 shows images captured of GFP-HUVECs growing in the off-chip fibrin hydrogel clot after 48 and 96 hours. The effects of increased TCM concentrations can be observed in the development of more advanced vessel-like structures. After 96 hours, cells were immunostained for integrin α_*v*_β_3_ and analysed using flow cytometry. HUVECs and NHLFs could be distinguished from one another due to the GFP HUVEC emission. Immunofluorescent α_*v*_β_3_ integrin intensity values for each cell type were plotted as histograms and a peak fitting function was used to find the modal emission intensity. This allowed for the expression of α_*v*_β_3_ to be accurately determined for each EGM : TCM ratio.

**Fig. 5:**
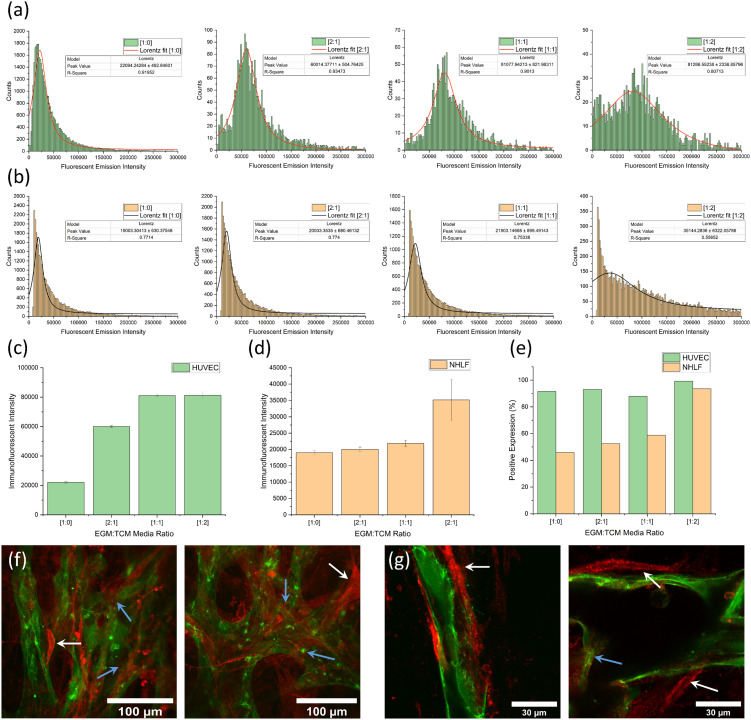
Flow cytometry data for fibrin-containing cultures showing (a) a series of graphs showing histogram plots and peak fits of α_*v*_β_3_ integrin immunofluorescent emission intensities from GFP-HUVECs. (b) A series of graphs showing histogram plots and peak fits of α_*v*_β_3_ integrin immunofluorescent emission intensities from NHLFs. The lowest TCM concentrations ([1 : 0]) are shown on the left and highest TCM concentrations ([1 : 2]) on the right. (c) A graph of peak immunofluorescent intensities for GFP-HUVECs. An increase of 430% was observed from the no-TCM [1 : 0] value (22 000 ± 500) to the highest [1 : 2] TCM value (81 000 ± 2000). (d) A graph of peak immunofluorescent intensities of NHLFs. An increase of 75% was observed from the no-TCM [1 : 0] value (19 000 ± 300) to the highest [1 : 2] TCM value (35 000 ± 6000). (e) A graph showing the percentage of HUVECs and NHLFs positive for a α_*v*_β_3_ integrin expression for the same range of [EGM : TCM] ratios. The vast majority (90%+) of HUVECs were positive regardless of TCM concentration whereas NHLFs show an increase in cells positive for α_*v*_β_3_ integrin expression from 45% up to 85%. Immunofluorescent staining images (red) of integrin expression in healthy [1 : 0] on-chip vasculature cultures with 40× (f) and 100× (g) objectives. White and blue arrows highlight NHLF and HUVEC cell immunofluorescent for integrin α_*v*_β_3_ expression, respectively.

Fig. 5a and b show histogram plots for each of the EGM : TCM media ratios for HUVEC and NHLF, respectively. A trend of increasing modal emission intensity can be observed with increasing TCM concentrations for each cell type – indicating a TCM-induced α_*v*_β_3_ upregulation. Fig. 5c and d show plots of peak intensity values for each media ratio, for HUVEC and NHLF, respectively. A substantial 290% increase in expression intensity was observed between HUVECs grown in media without TCM ([1:0]) and media grown using a [2 : 1] EGM : TCM media ratio ((6 ± 0.05 × 10^4^) compared to (2.2 ± 0.05 × 10^4^). This further increased to 430% when the highest TCM concentration was used, giving a peak intensity of (8.1 ± 0.2 × 10^4^). In contrast, NHLF expression intensity was relatively unchanged in response to [2 : 1] and [1 : 1] EGM : TCM media, however, an increase of 75% (up to 3.5 ± 0.6 × 10^4^) was observed with a [1 : 2] EGM : TCM media ratio. Calculation of the percentage of cells positive for α_*v*_β_3_ expression (Fig. 5e) revealed that on average, 92% of HUVECs were positive for α_*v*_β_3_ expression regardless of TCM concentration. This was expected, as HUVECs will be in an angiogenic state and should therefore be expressing this integrin. Observation of NHLF expression showed that approximately 45% were initially positive for α_*v*_β_3_ expression which was then observed to increase dramatically up to 94% when a [1 : 2] media ratio was used. The large increase observed with [1 : 2] media could potentially have been due to the TCM concentration being sufficient to induce fibroblasts to display a cancer-associated phenotype and increase integrin α_*v*_β_3_ expression.^30^

Overall, flow cytometry results throughout this fibrin-containing angiogenesis assay suggest that the presence of TCM results in the upregulation of integrin α_*v*_β_3_. As this integrin is directly associated with angiogenesis, it is reasonable to suggest that TCM facilitates increased rates of angiogenesis compared to EGM media alone. In addition to flow cytometry, immunostaining was also performed using on-chip vasculature cultures to observe α_*v*_β_3_ integrin expression across the network. As shown in Fig. 5f and g, integrin α_*v*_β_3_ (red) appeared to be expressed on the surface of endothelial cells (blue arrows) as well as by the supporting NHLF cells (white arrows), the crescent-shaped cells surrounding the vessels. This was consistent with flow cytometry observations along with previous observations throughout literature, which have observed integrin expression in supporting stromal cells due to their binding to similar ECM proteins as vascular endothelial cells.^27,28^

Results thus far have demonstrated the ability of TCM-conditioning to influence vasculature to display tumour vasculature-like properties. Analysis of vessel morphology and integrin protein expression has shown that networks conditioned with TCM show characteristics that align with those observed in *in vivo* tumour vasculature. Experimentation, therefore, progressed to determining the efficacy of using integrin α_*v*_β_3_-targeted LS to improve tumour vasculature targeting, and further investigate novel methods of delivering targeted LS to tumours using MB-mediated sonoporation. Results from this investigation would not only demonstrate the potential of integrin α_*v*_β_3_ targeting and MB delivery but also demonstrate the effectiveness of this tumour vasculature model as a tool for evaluating potential treatments.

### Perfusion of integrin α_*v*_β_3_-targeted liposomes

This series of experiments focused on evaluating the effectiveness of targeting LS to integrin α_*v*_β_3_, through observation of LS accumulation in tumour vasculature networks. Observing an increase in LS accumulation in networks displaying tumour-vasculature-like properties compared to healthy vasculature would demonstrate the potential of α_*v*_β_3_ as a target for tumour vasculature. 2 μg mL^−1^ and 4 μg mL^−1^ antibody concentrations were each tested to observe whether an increase in the targeting moiety increased accumulation – providing evidence that the inclusion of α_*v*_β_3_ targeting was driving LS accumulation. Isotype control antibodies were also tested at each concentration to observe whether any increased accumulation was a result of non-specific antibody binding, as opposed to binding specifically to integrin α_*v*_β_3_. Isotype antibodies therefore show if an increase in fluorescent signal is due to an increased targeting efficacy or merely an artifact from increased non-specific binding. Biotin -neutravidin binding was used to bind biotinylated α_*v*_β_3_ antibodies onto the surface of red fluorescent (DOPE-Atto647N) LS containing Biotin-PEG2000, as described in section 2.4. This method of binding antibodies to the surface of MBs and LSs has been well documented and used similar ratios to many previous studies.^36,40,54,67^

**Fig. 6:**
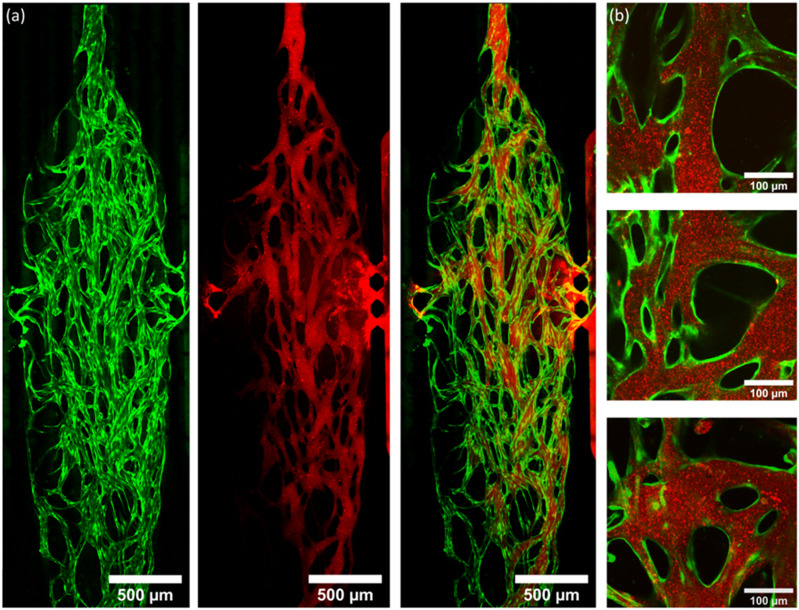
(a) Tile-scanned images of vasculature networks (green) and LS perfusion (red) alongside an overlayed image of both channels, taken using a 10× objective. LS were observed to enter the chamber from the high pressure side channel (right) and perfuse across the network. (b) High magnification images (50× objective) showing LS perfusing exclusively through networks and remaining confined within the vessels. Images were taken from multiple chips used throughout this investigation.

**Fig. 7:**
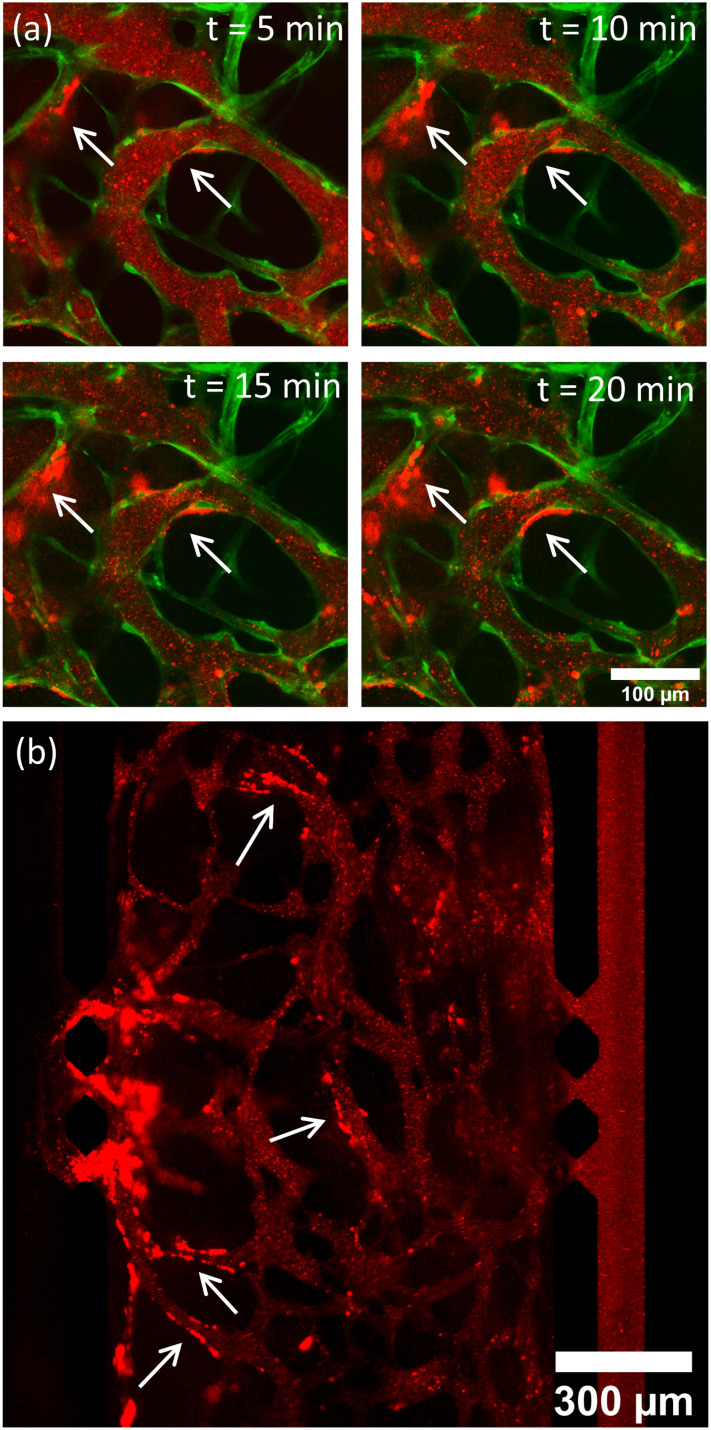
(a) Time lapse image, taken in 5 minute intervals of targeted LS (red) accumulating on the surface of vasculature (green) grown using [2 : 1] EGM : TCM media. (b) Low magnification (10×) image taken of LS accumulation after the majority of LS had perfused through the network. White arrows indicate regions where accumulation is observable.

**Fig. 8:**
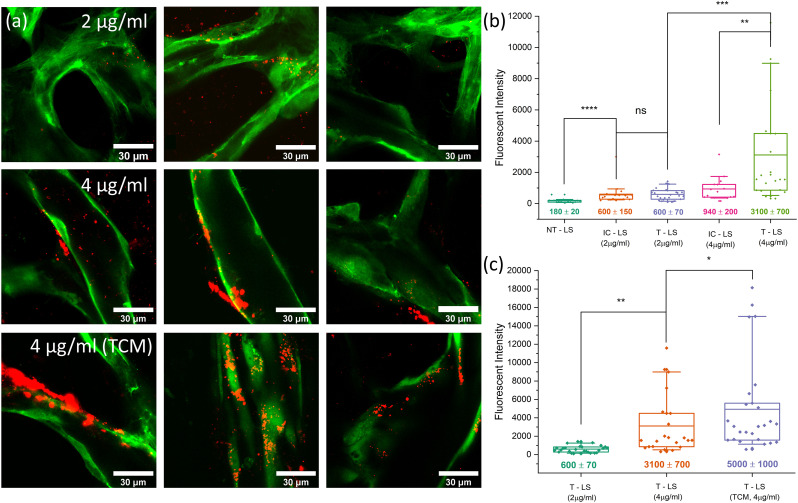
(a) High magnification post-perfusion images showing LS (red) accumulation after 2 μg ml^−1^ and 4 μg ml^−1^ targeted LS have perfused through healthy networks and 4 μg ml^−1^ targeted LS have perfused through TCM-conditioned networks. Image slices were taken as part of *z*-stacks which covered each vessel in1.5 μm slices with a 100× oil-immersion objective. (b) A boxplot graph showing quantified LS accumulation for non-targeted LS controls (NT–LS), 2 μg ml^−1^ targeted LS (T–LS), 4 μg ml^−1^ targeted LS (T–LS) and isotype controls (IC–LS) for the both targeting concentrations. 2 μg ml^−1^ targeting showed no significant increase compared to that of the same isotype control experiment whereas 4 μg ml^−1^ showed a 3-fold increase in LS intensity compared to 4 μg ml^−1^ isotype controls. (c) A boxplot graph showing quantified LS accumulation values for 2 μg ml^−1^ and 4 μg ml^−1^ targeted LS perfusions in healthy networks (also shown in (b)) alongside 4 μg ml^−1^ targeted LS perfusions through networks conditioned with TCM.

Fig. 6 shows images taken of LS perfusing through a network, with an overlayed image showing how LS are confined exclusively within the vessels. Flow throughout each network was generally homogeneous and the majority of the vessels were perfused with LS. Regions without flow were occasionally observed and suspected to be a result of insufficient anastomosis, creating backpressure in the vessels due to the lack of an outlet. Regions without flow were identified and excluded from accumulation imaging. Whilst LS were confined within the vessels, some leakage was observed at the chamber entrance. This is due to the lack of adhesion between PDMS and endothelial cells at the pillar gaps. Coating the side channels with laminin and basement membrane extract (BME) improved cell adhesion but did not eliminate leakage. To prevent this from affecting results, regions of vasculature located within 100 μm of the pillar gap entrance were not imaged for accumulation measurements. Evidence of targeted LS accumulation could be observed in time-lapse images taken whilst LS were perfusing through networks. Fig. 7a shows a series of images taken in 5 minute intervals of liposomes perfusing through vasculature (grown using [2 : 1] EGM : TCM media) with white arrows highlighting regions in which LS were appearing to accumulate on the vessel surface. Fig. 7b also shows a lower-magnification image of a [2 : 1] vasculature system, taken after most LS had perfused throughout the network. LS can be observed to be accumulating across the surface of various vessels, primarily those with smaller diameters. This was not initially expected, as bead tracking experiments had shown that flow through smaller diameter vessels was faster than that through larger vessels, therefore indicating higher rates of shear stress in smaller vessels. On the other hand, integrin α_*v*_β_3_ is known to be expressed more highly on vessels undergoing higher rates of flow due to the increased intramural pressure created by the reduced vessel diameters.^68^ As described in section 2.5, LS were perfused for an hour before fresh media was added to the inlet reservoir. A further hour was then given for media to perfuse the network and any remaining unbound LS are washed away. Imaging was then performed across several regions of the chips, using a 100× objective and *z*-stacks to capture entire profiles of vessels. Accumulation was quantified using the particle analysis tool in ImageJ. The average intensity was calculated for each *z*-stack then plotted as a single data point in a boxplot. Fig. 8a shows typical post perfusion image slices taken after the perfusion of 2 μg mL^−1^ and 4 μg mL^−1^ targeted LS through healthy networks, and 4 μg mL^−1^ targeted LS through TCM conditioned networks. The graphs alongside these images, shown in Fig. 8b and c, show fluorescent intensity values determined from the post perfusion images, where each data point on the boxplot corresponds to a single z-stack.

Non-targeted LS (NT-LS), LS with the same lipid composition but without any antibodies present, showed very little accumulation within the vasculature networks and resulted in an average fluorescent intensity of 180 ± 20. Targeted LS conjugated with 2 μg mL^−1^ α_*v*_β_3_ antibody (T-LS 2 μg mL^−1^) resulted in an average accumulation intensity of 600 ± 70, showing a significant increase compared to the previous condition. LS were observed to accumulation diffusely throughout the vasculature, not appearing to bind to any regions specifically. Perfusions performed with LS conjugated with 2 μg mL^−1^ isotype antibody (IC-LS 2 μg mL^−1^), found a similar degree of accumulation (600 ± 120), suggesting the increase in adhesion is primarily due to the non-specific binding of antibodies to the vessel walls. Perfusion of LS targeted with 4 μg mL^−1^ α_*v*_β_3_ antibodies (T-LS 4 μg mL^−1^) was previously observed to accumulate on the surface of the vasculature as perfusion occurred (Fig. 6). This increase was also reflected when accumulation was quantified, with the average intensity found to be 3100 ± 600 – a 5-fold increase on the 2 μg mL^−1^ LS accumulation average. Several regions imaged and analysed were found to contain large numbers of LS bound to specific regions of the vasculature, observable in Fig. 8a.

These regions resulted in fluorescent intensity values of typically 2000 or above. Regions analysed below this value were found to show accumulations more consistent with non-specific antibody binding. This was reflected in the 4 μg mL^−1^ isotype control (IC-LS 4 μg mL^−1^) results which also showed an increase in accumulation compared to 2 μg mL^−1^ perfusions but did not result in regions of significant LS binding. This, therefore, suggests that several of the lower value intensities observed for T-LS (4 μg mL^−1^) perfusions were due to non-specific binding, whereas accumulations above 2000 were due to specific α_*v*_β_3_ integrin binding. Performing a Mann–Whitney *U*-test revealed no statistical significance between 2 μg mL^−1^ isotype or targeted LS accumulations, whereas 4 μg mL^−1^ targeting showed ** (*p* < 0.01) significance when tested against the 4 μg mL^−1^ isotype control. As the accumulation results from perfusions using LS targeted with 2 μg mL^−1^ did not indicate that this concentration of antibody was having an impact on targeting, it was decided that experiments moving forwards would only use LS targeted with the higher 4 μg mL^−1^ α_*v*_β_3_ antibody concentration. LS were now perfused through TCM-conditioned networks to observe whether upregulation of integrin α_*v*_β_3_ resulted in increased rates of LS accumulation. Post perfusion imaging, performed after an hour of washing and shown in Fig. 8a, revealed an apparent increase in both the magnitude and frequency of regions of high LS accumulation. This increase was reflected in intensity values, which saw an average increase in LS accumulation of 61% compared to healthy network perfusions at the same targeting concentration. The maximum intensity values were observed to increase to over 18 000 within [2 : 1] EGM : TCM networks, an increase of 50% of the previously observed maximum value of approximately 12 000. Furthermore, many of the accumulation values were found to exceed the previously supposed threshold for specific targeting of 2000. This increase in the overall distribution of TCM accumulation values can be best observed when comparing the median intensity values for each distribution. Healthy network median intensity values were calculated to be 1500 ± 800 whereas [2 : 1] EGM : TCM network values were found to be 3000 ± 1200 – showing a consistent increase across the entire accumulation intensity range. Considering the previously observed upregulation of integrin α_*v*_β_3_ in similar off-chip culture models, this suggests that integrin α*_v_*β_3_ may be being expressed more consistently and homogeneously across [2 : 1] EGM : TCM networks. Together with the observations of TCM network morphologies, suggests that this may be a result of the network being induced to remain in an angiogenic state rather than stabilise into mature, healthy vessels. The disorganised, tortuous network structures produced through [2 : 1] EGM : TCM conditioning may also have influenced intensity values by causing irregular flow and presenting locations in which liposomes could readily accumulate. Together, the increase in median and maximum LS accumulation values with [2 : 1] EGM : TCM conditioned networks agrees well with flow cytometry observations which observed an increase in integrin α_*v*_β_3_ expression intensity as well as an increase in the number of cells positive for integrin expression. Whilst a series of follow-up experiments using drug-loaded LS is required to further quantify the therapeutic effect of using integrin α_*v*_β_3_ targeting, these results provide strong evidence that integrin α_*v*_β_3_ targeting could facilitate the increased accumulation of LS in tumour-associated vasculature.

### Microbubble-mediated delivery of α_*v*_β_3_-targeted LS

To investigate the use of MBs as a potential method of improving the delivery of targeted LS to tumour vasculature, 4 μg mL^−1^ targeted LS were conjugated to the surface of MBs again using biotin–neutravidin binding and incorporating biotinylated DSPE-PEG-2000 lipids into MBs. This formed what is referred to as targeted LS–MB conjugates (T–LS–MB). Along with the complete exposure condition of T–LS–MBs perfused through TCM networks then burst with US, two further control conditions were tested. The first control looked to observe the impact of bursting the MBs with US by allowing T–LS–MBs to perfuse through the networks without the use of an US trigger. Differing LS accumulation intensities would then demonstrate the impact of MB-mediated sonoporation on LS deposition within the vasculature. The second control condition sought to observe the impact of using targeted conjugates by exposing healthy networks to the complete, T–LS–MBs and US exposure condition. As MBs are significantly larger than LS and their increased surface area will make them more susceptible to shear stresses from fluid flow. Increased adhesion will therefore be required for MBs to remain successfully bound to the vessel surface compared to LS. This may affect the previously observed targeting efficacy found when using 4 μg mL^−1^ integrin α_*v*_β_3_ antibodies.

**Fig. 9:**
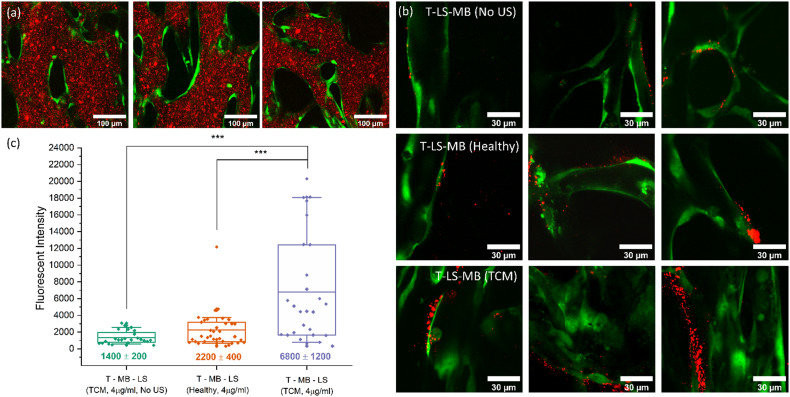
(a) Mid-perfusion images taken of T-LS-MB conjugates perfusing through vasculature. (b) Post-perfusion images taken of fluorescent LS accumulation for each T-LS-MB experimental condition. Image slices were again taken as part of *z*-stacks which covered each vessel in 1.5 μm slices with a 100× oil-immersion objective. (c) A boxplot showing quantified LS accumulation intensities for T–LS–MB exposures in TCM networks with and without US, as well as in healthy networks with US.

Perfusion experiments were repeated in the same configuration previously used with US now being incorporated. Microfluidic chips were insonated after MBs were observed to fully populate the vasculature networks. Fig. 9a shows images taken of T–LS–MB perfusing through the network. Conjugating LSs to MBs results in a much larger fluorescent particle size when compared with fluorescent LS perfusions previously shown in Fig. 5. Due to the limitations of the US/imaging setup, it was difficult to capture images of MBs post-US exposure, as MBs would repopulate the network before images could be acquired. Fig. 9b shows post perfusion images of LS accumulation alongside quantified fluorescent intensity values for each experimental condition (Fig. 9c). Images shown of accumulation shown of liposome accumulation in T–LS–MB (No US) conditions correspond to measured intensities of approximately 1000, whereas T–LS–MB (TCM) images show accumulations corresponding to intensities of 6000 and above. Results, in this case, were not compared to previous LS-only perfusions as it is difficult to equate the total number of LS perfusing throughout the networks. Whilst the same total number of LS are added to the reservoirs, many of the larger MBs rise to the top of the reservoirs or stick to the top of the inlet channels before they are able to perfuse through the network. It would therefore be inaccurate to equate the accumulation values.

Observation of LS accumulation after perfusion of T–LS–MBs in TCM networks without an US trigger found relatively few LS across the vessel surfaces. Regions of LS accumulation were commonly observed however the magnitude of LS accumulation in each region was much lower than previously observed. The high frequency of LS regions suggests that integrin α_*v*_β_3_ is being expressed regularly throughout the network, agreeing with previous TCM conditioning observations. However, the low-intensity values suggest that minimal numbers of LS were free to accumulate within the networks. This shows that a lack of US trigger prevents significant numbers of LS from being released from the MB surface. It is difficult to determine whether the use of 4 μg mL^−1^ targeting was sufficient to allow for MB adherence to the vessel surface as it is possible that some regions of LS accumulation are a result of MB accumulation and subsequent dissolution. However, the low intensities would suggest that targeting adhesion is insufficient in allowing significant numbers of MBs to persist within the network. Alternatively, low numbers of unbound LS in the MB solution may be resulting in the observed accumulation intensities, which would result in the frequent observation of low numbers of LS accumulated within the vasculature.

Perfusion and subsequent bursting of T–LS–MB conjugates in healthy vasculature networks were found to result in significantly increased numbers of LS compared to the previous control condition. LS were found to be distributed across many regions of the vasculature surface, resulting in an average fluorescent intensity value of 2200 ± 400. Despite these perfusions being performed in healthy networks, this intensity value was higher than the 1400 ± 200 value found after perfusions through [2 : 1] EGM : TCM networks without an US trigger. This demonstrates the impact of bursting MBs on the extent of LS accumulation. Finally, exposing networks to T–LS–MBs in TCM networks with an US trigger was observed to significantly increase LS accumulation. Post perfusion imaging observed large swathes of LS across several points on the vessel surface, consistent with observations made throughout 4 μg mL^−1^ targeted LS perfusions. Quantification of accumulations found an average intensity of 6800 ± 1200, a 3-fold increase in intensity compared to the same perfusion performed in healthy vasculature. This result provided compelling evidence that the inclusion of 4 μg mL^−1^ integrin α_*v*_β_3_ antibodies successfully facilitated the targeting of MB-LS conjugates and allowed for significantly improved LS delivery when MBs were burst with US. Intensity values for this complete exposure condition were found to consistently exceed control values, resulting in a high *** (*p* > 0.001) statistical significance value when subject to a non-parametric Mann–Whitney *U*-test. Images taken after US exposure were unable to observe any immediate effects of MB sonoporation on endothelial integrity using this current imaging and experimental setup. The use of membrane permeability probes such as propidium iodide would improve the ability to identify any sonoporation effects on endothelial cell membranes. Observation of the opening of gap at endothelial cell–cell contacts was not observed in this study in contrast to observations made throughout previous studies.^36,41^ However, the imaging modalities and timescales used throughout this study differ significantly from those used previously. A worthwhile follow-up experiment could employ the use of a cell detachment agent, such as EDTA, to induce cell detachment and contact opening to serve as a positive control.

It is also suggested that the lack of gap formation may be a consequence of using a physiologically accurate vasculature model which is complete with supporting ECM and stromal components. Observations of junction opening are often made in systems comprising of endothelial cells seeded onto polycarbonate or PDMS surface, which minimally supports the adhesion of cells compared to a functional basement membrane layer. In contrast to this, many *in vivo* studies have observed the opening of the blood brain barrier (BBB) with the use of MBs and focused US, suggesting the integrity of the endothelium is affected significantly by sonoporation.^69,70^ A study by Tung *et al.* observed that the BBB was only opened by non-linear MB oscillation when the MB diameter was similar to the capillary diameter but in all instances of inertial cavitation.^70^ As the vessel diameters in the vasculature networks in this study were more comparable to precapillary vessels (>25 μm) than capillaries (<10 μm), this may explain the lack of MB impact on cell–cell junction integrity. Continued vasculature integrity after US and MB exposures suggested that any events resulting in irreversible sonoporation were not sufficient enough to compromise precapillary vessel function. However, whilst the overall vascular integrity appeared unaffected by MBs and US, the transient opening of cell–cell contacts by MB sonoporation was not investigated. A potential mechanism for the increased accumulation of LS conjugated to MB is the sonoprinting of LS onto the surface of the endothelium. De Cock *et al.* and Roovers *et al.* first described the process of sonoprinting as a mechanism by which nanoparticle-loaded MB facilitate the deposition of nanoparticles onto 2D cell monolayers and tumour spheroids.^71,72^ In spheroids, this allowed for the increased release of drugs from nanoparticles attached to the outer layers of the spheroids. These observations are also consistent with previous observations by Bourn *et al.*, which observed the increased deposition of doxorubicin-loaded LS when conjugated to MBs – increased drug accumulation and decreased spheroid viability was observed as a result.^40^ Several follow up experiments could be performed to further investigate the MB-related phenomena observed throughout this study and previous studies. It is suggested that very similar experiments previously conducted on simplistic 2D systems could be repeated using this system to observe the impact of improving the physiological relevance of the system. Propidium iodide, dead cell staining, and calcium fluctuations could all be implemented to probe the effects of MBs on cell–cell contacts, vessel permeability and overall endothelial integrity.^73^

Whilst the use of indirect tumour conditioning in this study prevented the direct evaluation of tumour cell viability, increased accumulation, and uptake of therapeutics in the vasculature can still be used as an indicator of improved drug delivery. The use of TCM conditioning also enabled the routine production of tumour-associated vasculature compared to previous tumour vasculature systems which have relied on cancer cell-vasculature co-cultures, demonstrating the advantages of this relatively simplistic system.

## Conclusion

This study presents a novel method of producing perfusable vasculature cultures which display tumour-associated properties. Characterisation of vessel area, diameter, and integrin α_*v*_β_3_ expression revealed properties consistent with that observed in *in vivo* tumour vasculature. The microfluidic system developed to culture vasculature allowed for perfusion of model therapeutics exclusively through the vessels, with rates of flow and shear stress similar to those found within capillaries. Targeting LS with 2 μg mL^−1^ integrin α_*v*_β_3_ antibodies was found to result in similar rates of accumulation compared to isotype controls of the same concentration, suggesting this concentration was not significant enough to facilitate specific targeting. Whereas 4 μg mL^−1^ targeted-LS were found to result in increased rates of LS accumulation, revealing a 3-fold increase compared to 4 μg mL^−1^ isotype controls. Perfusion of 4 μg mL^−1^ targeted-LS through [2 : 1] EGM : TCM networks observed a further 60% increase in accumulation intensities compared to healthy networks. Together with the observation of TCM-induced integrin α*_v_*β_3_ upregulation in off-chip vasculature cultures, provides evidence that TCM induced α_*v*_β_3_ upregulation facilitates the uptake of targeted LS in microfluidic vasculature networks. The destruction of T-MB-LS within the vasculature was observed to significantly increase LS accumulation in TCM networks compared to healthy networks, finding a 3-fold increase. Overall, these results indicated that the delivery of drug-loaded LS to tumours could be enhanced through the combinatory use of integrin α_*v*_β_3_ targeting and MB-mediated LS delivery. Whilst initial results appear promising, several additional experiments are still required to further elucidate the efficacy of integrin targeting and MB-mediated drug delivery.

Together, experiments throughout this study have demonstrated the ability of this microfluidic vasculature system to evaluate potential anti-cancer therapeutics.

## Data availability

The raw data required to reproduce these findings are available to download from https://doi.org/10.5518/1092. The processed data required to reproduce these findings are available to download from https://doi.org/10.5518/1092.

## Conflicts of interest

There are no conflicts of interest to declare.

## Supplementary Material

LC-023-D2LC00963C-s001
