# Supplementary material for: Tumour associated vasculature-on-a-chip for the evaluation of microbubble-mediated delivery of targeted liposomes
Source: Lab Chip. 2023 Feb 6;23(6):1674–93. doi: 10.1039/d2lc00963c (PMC10013341; doi:10.1039/d2lc00963c)
Supplement: LC-023-D2LC00963C-s001 [file LC-023-D2LC00963C-s001.pdf]

This journal is © The Royal Society of Chemistry 2023

Supplementary Information

Hydrostatic Flow Equations:

Flow rate, Q(t) is equal to the pressure drop across the channel, ΔP(t) divided by the hydraulic resistance, R_{hyd}:

$$Q(t) = \frac{\Delta P(t)}{R_{hyd}}$$

ΔP(t) consists of a gravitational pressure term which is dependent on the height difference between inlet and outlet reservoirs, ΔH(t) and a capillary pressure term, P_{cap} which is dictated by the contact angles created by fluid within the reservoirs:

$$\Delta P(t) = \rho g \Delta H(t) - P_{cap}$$

R_{hyd} can be derived using fundamental fluid dynamics using the Hagen-Poiseulle and Navier-Stokes equations. It is found to be dependent on channel length, L, width, w and height, h:

$$R_{hyd} = \frac{12\eta L}{h^3 w}\left(1 - \frac{0.63h}{w}\right)^{-1}$$

Due to the simultaneous emptying and filling of reservoirs, the flow rate was found to decrease exponentially with time:

$$Q(t) = Q(t_0)\exp\left(-\frac{t}{\tau}\right) \quad \text{where,} \quad \tau = -\frac{R_{hyd}\pi r^2}{2\rho g}$$

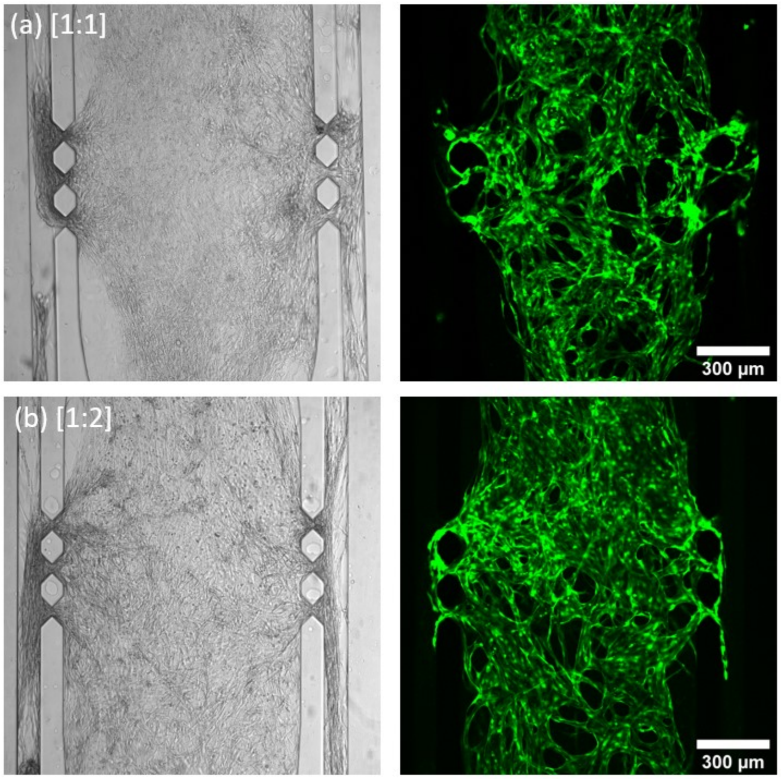

Supplementary figure 1 – Bright field (left) and fluorescent confocal images (right) taken of vasculature networks after 7 days, grown using (a) [1:1] and (b) [1:2] media.

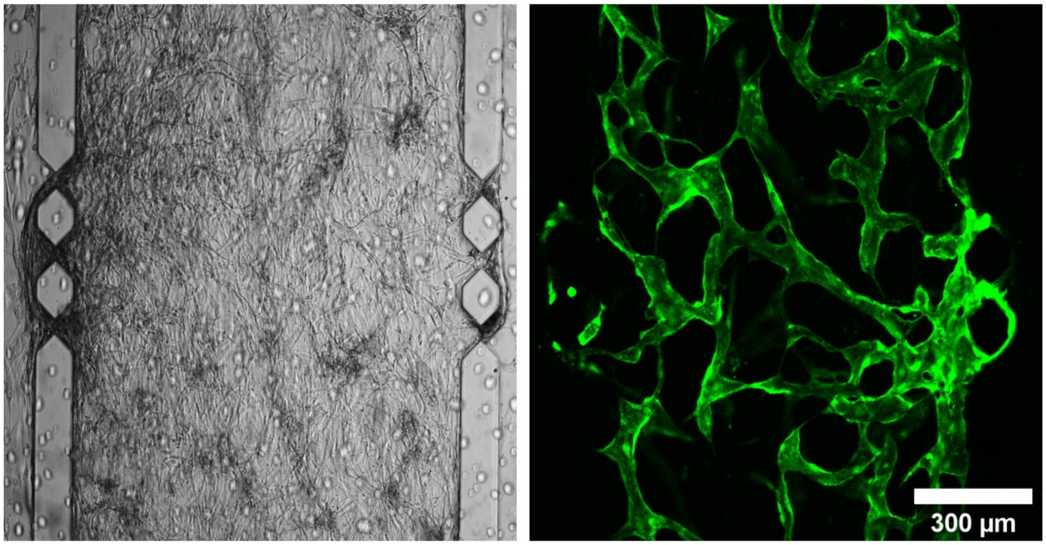

Supplementary figure 2 - Bright field (left) and fluorescent confocal images (right) taken of a vasculature network grown for 7 days using EGM-2 complete with VEGF and FGF.

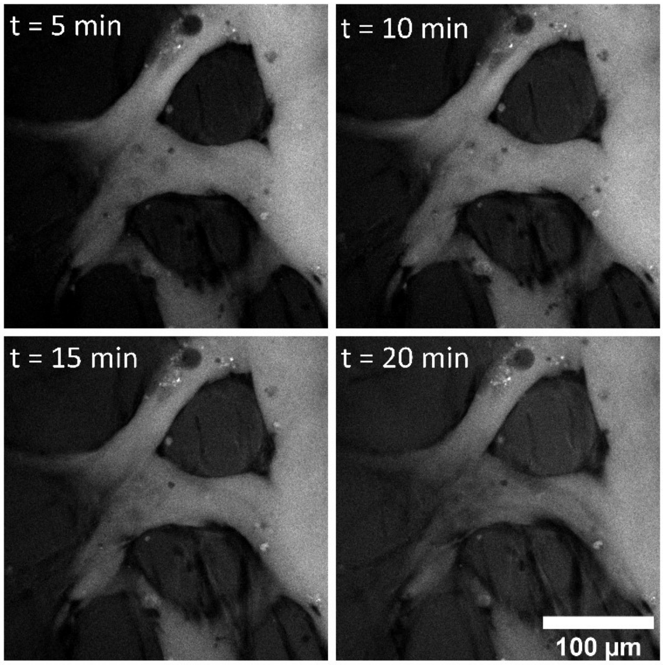

Supplementary figure 3 – Time lapse images of fluorescent Dextran leakage through vessel walls.

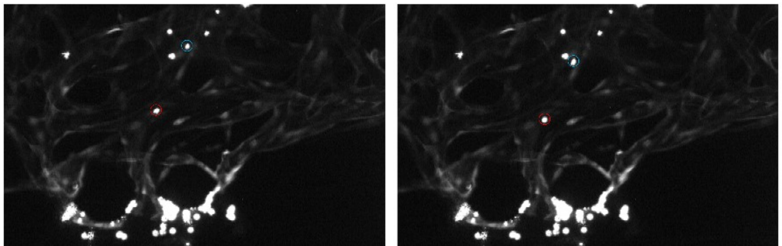

Supplementary figure 4 – Video frames showing fluorescent beads traversing a vasculature network. Bead trajectories were tracked and used to calculate intramural flow rates throughout the networks.

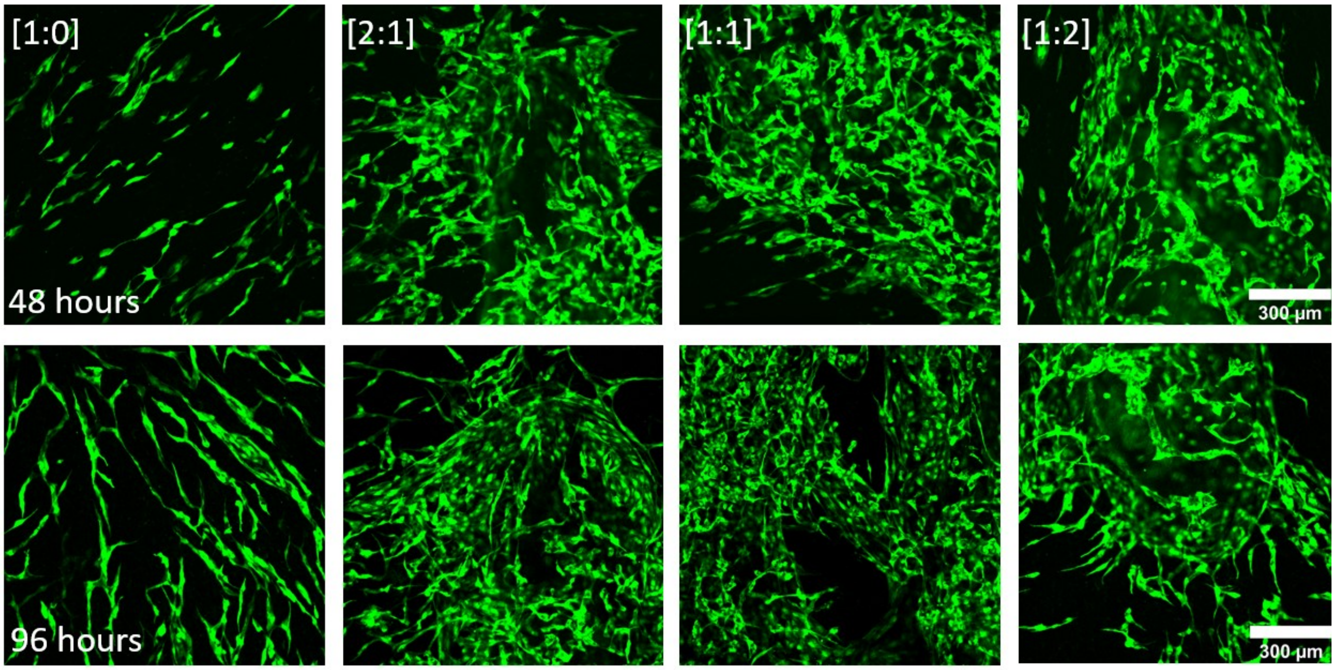

Supplementary figure 5 – Confocal images taken of GFP-HUVECs (suspended in fibrin) branching into vessel-like structures. The effects of increasing EGM:TCM concentrations can be seen in the development of more advanced vessel-like structures after 96 hours.
